# T Cell-Specific Overexpression of Acid Sphingomyelinase Results in Elevated T Cell Activation and Reduced Parasitemia During *Plasmodium yoelii* Infection

**DOI:** 10.3389/fimmu.2019.01225

**Published:** 2019-05-31

**Authors:** Matthias Hose, Anne Günther, Hanna Abberger, Salina Begum, Marek Korencak, Katrin A. Becker, Jan Buer, Astrid M. Westendorf, Wiebke Hansen

**Affiliations:** ^1^Institute of Medical Microbiology, University Hospital Essen, University Duisburg-Essen, Essen, Germany; ^2^Institute of Molecular Biology, University Hospital Essen, University Duisburg-Essen, Essen, Germany; ^3^Institute for HIV Research, University Hospital Essen, University Duisburg-Essen, Essen, Germany

**Keywords:** sphingolipids, acid sphingomyelinase, T cells, T cell activation, malaria

## Abstract

The enzyme acid sphingomyelinase (ASM) hydrolyzes sphingomyelin to ceramide and is thereby involved in several cellular processes such as differentiation, proliferation, and apoptosis in different cell types. However, the function of ASM in T cells is still not well characterized. Here, we used T cell-specific ASM overexpressing mice (t-ASM/CD4cre) to clarify the impact of cell-intrinsic ASM activity on T cell function *in vitro* and *in vivo*. We showed that t-ASM/CD4cre mice exhibit decreased frequencies of Foxp3^+^ T regulatory cells (Tregs) within the spleen. Enforced T cell-specific ASM expression resulted in less efficient induction of Tregs and promoted differentiation of CD4^+^CD25^−^ naïve T cells into IFN-γ producing Th1 cells *in vitro*. Further analysis revealed that ASM-overexpressing T cells from t-ASM/CD4cre mice show elevated T cell receptor (TCR) signaling activity accompanied with increased proliferation upon stimulation *in vitro*. *Plasmodium yoelii* infection of t-ASM/CD4cre mice resulted in enhanced T cell activation and was associated with reduced parasitemia in comparison to infected control mice. Hence, our results provide evidence that ASM activity modulates T cell function *in vitro* and *in vivo*.

## Introduction

The sphingolipid metabolism involves several enzymes including acid sphingomyelinase (ASM), a lipid hydrolase enzyme constitutively expressed in lysosomes and released to the outer leaflet of the cell membrane upon triggering of e.g., CD95, TNF receptors, or CD28 (1–4). ASM cleaves sphingomyelin into ceramide and phosphocholine (5), resulting in the formation of ceramide-rich signaling platforms at the outer leaflet of the plasma membrane (6), which play an important role in regulating differentiation, proliferation, and apoptosis in different cell types (7, 8). Studies performed with ASM-deficient mice or treatment of cells with ASM-inhibitors such as amitriptyline suggests that ASM activity is involved in T cell development and function (9–14). For CD8^+^ T cells, it was shown that ASM-deficiency results in impaired secretion of IFN-γ and cytotoxic granula in lymphocytic choriomeningitis (LCMV)-infected mice (11). Recently, two independent studies provided evidence that ASM activity is also involved in CD4^+^ regulatory T cell (Treg) development, survival, and function (12, 13). ASM-deficient or amitriptyline-treated mice had enhanced numbers of Tregs in comparison to wildtype (WT) or non-treated mice. In addition, ASM-deficient Tregs showed enhanced turnover of CTLA-4 and exhibited increased suppressive activity *in vitro* (13). Blocking of ASM activity in human CD4^+^ T cells by pharmacological inhibitors or by siRNAs has been shown to interfere with T cell receptor (TCR) signaling, proliferation, and T helper (Th) cell differentiation upon stimulation *in vitro* (14). However, most of these studies investigated the impact of ASM in the whole CD4^+^ T cell population or focused on Tregs, but did not investigate the impact of ASM on CD4^+^ non-Tregs. In addition, results from ASM-deficient mice do not exclude an indirect influence of other cells on T cell function, and treatment with ASM inhibitors might also act on other enzymes involved in the sphingolipid metabolism, such as acid ceramidase (15). Hence, the impact of cell-intrinsic ASM activity in CD4^+^ non-Tregs still remains unclear.

Malaria, caused by the parasite *Plasmodium*, is still one of the most deadly human infectious diseases worldwide. The parasite has a complex life cycle resulting in different innate and adaptive immune mechanisms involved in parasite control and clearance (16). During the blood-stage of infection, CD4^+^ T cells play a crucial role in regulating the immune response. While IFN-γ production by T cells and CD4^+^ T cell help for B cell responses are required for control and elimination of infected red blood cells (iRBCs) (17), CD4^+^Foxp3^+^ Tregs counteract excessive inflammatory immune responses that might result in exacerbated tissue damage. Expansion of Foxp3^+^ Tregs was observed in *Plasmodium*-infected patients (18, 19) as well as in different malaria mouse models (20–22) and their depletion resulted in enhanced T cell responses accompanied by reduced parasitemia (20). Besides Foxp3^+^ Tregs, IL-10 expressing CD4^+^ T cells with immunosuppressive function were described to be induced during *Plasmodium yoelii* (*P. yoelii*) infection (20, 23), at least in part due to stimulation of naïve T cells by IL-10 producing CD11c^+^ dendritic cells (DCs) (24). Hence, CD4^+^ T cells are important for the tight regulation of immune responses during *Plasmodium* infection.

In the present study, we provide evidence that T cell-intrinsic ASM activity is induced by anti-CD3/anti-CD28 stimulation. T cell-specific overexpression of ASM resulted in elevated phosphorylation of TCR signaling molecules and proliferative activity upon stimulation *in vitro*. Strikingly, *P. yoelii*-infected t-ASM/CD4cre mice exhibited a more activated T cell phenotype accompanied by improved pathogen clearance in comparison to infected control littermates. Thus, the sphingomyelin/ceramide pathway might represent a promising target for the modulation of T cell activity during ongoing immune responses *in vivo*.

## Materials and Methods

### Mice and Parasites

T-ASM mice (25) and CD4cre mice (26) both on C57BL/6 background were crossed and maintained under specific pathogen-free conditions at the Animal Facility of University Hospital Essen. Cryopreserved *P. yoelii* 17NXL (non-lethal) infected red blood cells (iRBCs) were passaged once through C57BL/6 wildtype mice before being used in experimental animals. For infection 1 × 10^5^ iRBCs were injected i.v. at day 0. The frequency of iRBCs (parasitemia) was determined by microscopic examination of Giemsa-stained blood films. All animal experiments were performed in accordance to the guidelines of the German Animal Protection Law and approved by the state authority for nature, environment, and customer protection, North Rhine-Westphalia, Germany.

### Cell Isolation and Activation

Single cell suspensions of splenocytes were generated by rinsing spleens with erythrocyte lysis buffer and washing with PBS supplemented with 2% FCS and 2 mM EDTA. T cells were isolated from splenocytes either by using the CD4^+^ or CD8^+^ T cell isolation kit (Miltenyi Biotec, Bergisch Gladbach, Germany) alone or followed by anti-CD4, anti-CD25, anti-CD8 staining, and cell sorting using an Aria II Cell Sorter (BD Biosciences, Heidelberg, Germany). T cells were stimulated with 5 μg/ml anti-CD3 plate-bound and 1 μg/ml anti-CD28 soluble (both BD Biosciences, Heidelberg, Germany) in IMDM culture medium supplemented with 10 % heat-inactivated FCS, 25 mM β-Mercapthoethanol and antibiotics (100 U/ml penicillin, 0.1 mg/ml streptomycin).

### T Cell Differentiation

For iTreg differentiation CD4^+^CD25^−^ T cells were stimulated with anti-CD3/anti-CD28 as described above in the presence of 20 ng/ml IL-2 (eBioscience, ThermoFisher Scientific, Langenselbold, Germany) and 5 ng/ml TGF-β1 (R&D Systems, Bio-Techne, Wiesbaden, Germany) for 72 h. Th1 cells were differentiated by stimulating sorted CD4^+^CD25^−^ T cells with anti-CD3/anti-CD28 in the presence of 200 ng/ml anti-IL-4 (eBioscience, ThermoFisher Scientific, Langenselbold, Germany) and 20 ng/ml IL-12 (R&D Systems, Bio-Techne, Wiesbaden, Germany) for 6 days. At day 3 cells were split and fresh IMDM medium supplemented with 1 μg/ml anti-CD28 and 200 ng/ml anti-IL-4 was added.

### Proliferation

T cells were labeled with the cell proliferation dye eFluor 670 (eBioscience, ThermoFisher Scientific, Langenselbold, Germany) according to the manufacturers protocol and stimulated for 3 days with anti-CD3 and anti-CD28 antibodies in the presence of irradiated splenocytes. Proliferation was assessed as loss of the proliferation dye by flow cytometry.

### Antibodies and Flow Cytometry

Anti-CD4, anti-CD8, anti-CD25, anti-IFN-γ (all BD Biosciences, Heidelberg Germany), anti-Foxp3, anti-Ki67 (eBioscience, ThermoFisher Scientific, Langenselbold, Germany), anti-Akt, anti-phosho-Akt(Ser473), anti-phospho-PLCγ1(Tyr783), anti-p38MAPK, and anti-phospho-p38MAPK(Thr180/Tyr182) (Cell Signaling, Frankfurt, Germany) were used as fluorescein isothiocyanate (FITC), pacific blue (PB), phycoerythrin (PE), BD Horizon V450, allophycocyanin (APC), AlexaFlour647 (AF647), PE-cyanin 7 (PE-Cy7), or peridinin-chlorophyll protein (PerCp) conjugates. Dead cells were identified by staining with the fixable viability dye eFluor 780 (eBioscience, ThermoFisher Scientific, Langenselbold, Germany). Intracellular staining for Foxp3 and Ki67 was performed with the Foxp3 staining kit (eBiocience, ThermoFisher Scientific, Langenselbold, Germany) according to the manufacturer's protocol. IFN-γ expression was measured by stimulating splenocytes with 10 ng/ml phorbol 12-myristate 13-acetate (PMA) and 100 μg/ml ionomycin (both Sigma-Aldrich, München, Germany) for 4 h in the presence of 5 μg/ml Brefeldin A, followed by treatment with 2% paraformaldehyd and 0.1% IGEPAL^®^CA-630 (Sigma- Aldrich, München, Germany), and staining with the respective antibody for 30 min at 4°C. For analyzing phosphorylation of TCR signaling molecules, cells were stimulated with 5 μg/ml anti-CD3 and 1 μg/ml anti-CD28 for 5 or 10 min, treated with 2% paraformaldehyd and 0.1% IGEPAL®CA-630, and stained with the respective antibody for 30 min at 4°C. Flow cytometric analyses were performed with a LSR II instrument using DIVA software (BD Biosciences, Heidelberg Germany).

### Serum Cytokines

Blood samples were collected, incubated at room temperature and centrifuged for 10 min at 6,797 × *g*. Cytokines were quantified by using a polystyrene bead-based Luminex Assay (R&D Systems, Abingdon, UK) and a Luminex 200 system with IS software according to the manufacturers recommendations.

### ASM Activity

CD4^+^ and CD8^+^ T cells were isolated from spleen by using the CD4^+^ or CD8^+^ T cell isolation kit (Miltenyi Biotec, Bergisch Gladbach, Germany). CD19^+^ B cells were isolated from splenocytes by cell sorting using an Aria II Cell Sorter (BD Biosciences, Heidelberg, Germany). T cells and B cells were either left untreated or stimulated with anti-CD3/anti-CD28 or 1 μg/ml LPS (Invivogen, Toulouse, France) overnight and lysed in 250 mM sodium acetate, 1% IGEPAL®CA-630 and 100 μM ZnCl_2_ for 5 min on ice, followed by bath sonication for 10 min. BODIPY FL 12C-SM (ThermoFisher Scientific, Langenselbold, Germany) in assay buffer was added to the cells to obtain 100 pmol SM and 0.1% NP40 within the reaction mix and incubated at 37°C with shaking. Lipids were extracted by adding chloroform/ methanol (2:1) and centrifugation, followed by isolation and drying of the lower phase. Cell pellets were resuspended in chloroform/ methanol (2:1) and spotted on a thin-layer chromatography plate. After running in chloroform/ methanol (80:20), plates were air-dried, scanned with a Typhoon FLA 9,500 laser scanner and analyzed with ImageQuant software (both GE Healthcare Life Sciences, US). Specific Asm activity was calculated as conversion of product per protein and time.

### Statistical Analysis

Statistical analyses were performed with Mann-Whitney *U*-test or two-way ANOVA with Bonferroni's multiple comparisons test. Statistical significance was set at the levels of ^*^p < 0.05, ^**^p < 0.01, and ^***^p < 0.001. All analyses were calculated with Graph Pad Prism Software (Graph Pad Software, La Jolla, CA).

## Results

### Decreased Relative Numbers of Foxp3^+^ Regulatory T Cells in T Cell-Specific ASM Overexpressing t-ASM/CD4cre Mice

**Figure 1:**
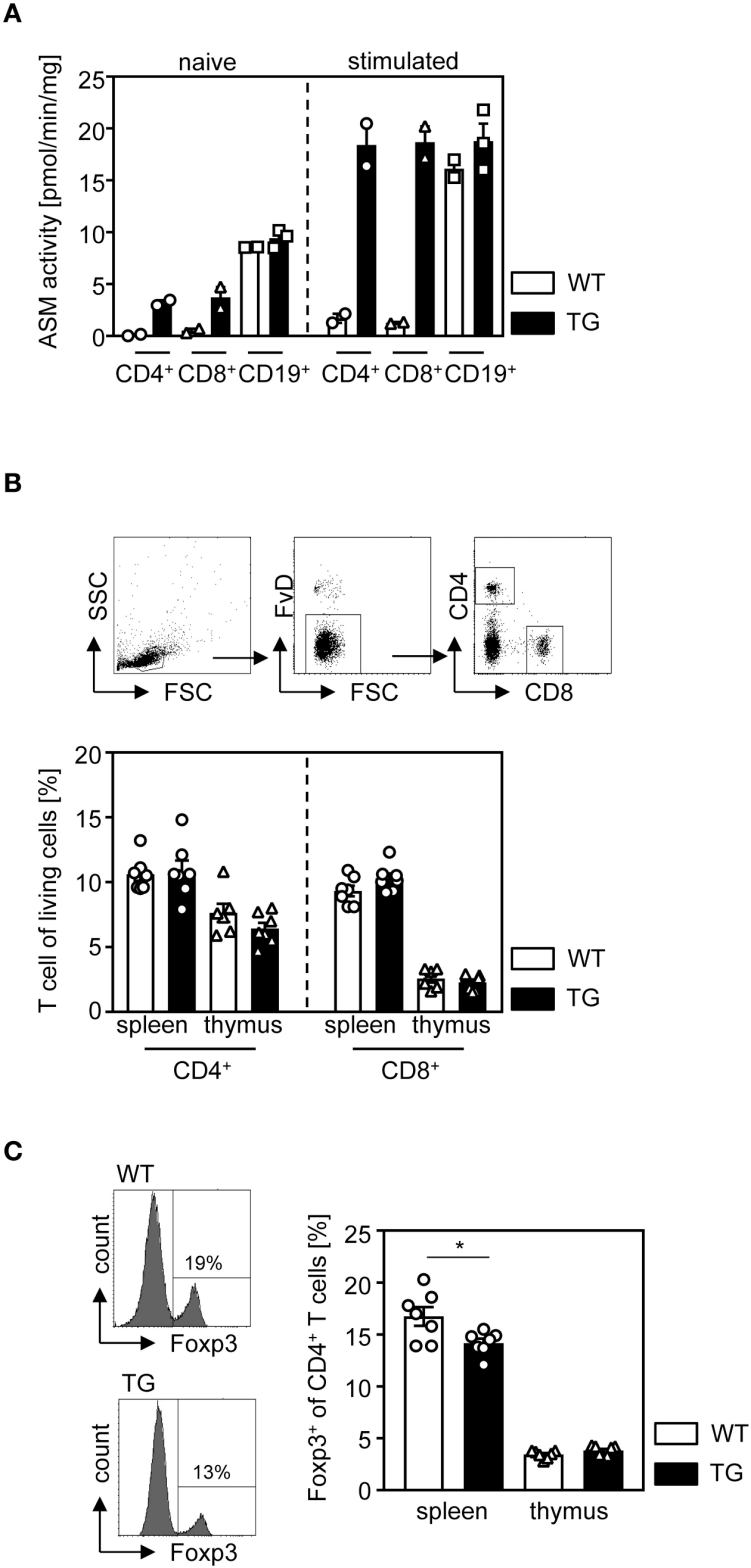
T cell-specific overexpression of ASM results in decreased relative numbers of regulatory T cells in the spleen. **(A)** CD4^+^ and CD8^+^ T cells as well as CD19^+^ B cells were isolated from t-ASM/CD4cre mice (TG) or t-ASM littermates (WT), left untreated or stimulated overnight with anti-CD3/anti-CD28 or LPS, respectively, and analyzed for ASM activity. **(B)** The percentages of CD4^+^, CD8^+^ T cells, and **(C)** Foxp3^+^ Tregs of CD4^+^ T cells within the spleen and thymus of t-ASM/CD4cre mice (TG) and t-ASM littermates (WT) were determined by flow cytometry. The gating strategy is shown in the upper panel of **(B)**. Data from **(A)** *n* = 2–3 mice and **(B,C)** two independent experiments with *n* = 6–7 mice in total are shown as mean ± SEM. *p < 0.05.

To study the cell intrinsic effect of ASM on the T cell phenotype, we made use of t-ASM mice crossed with CD4cre mice. In t-ASM mice, the *hprt* gene locus was replaced by a construct consisting of the ubiquitous CMV immediate early enhancer/chicken β-actin fusion promotor, and a loxP-STOP-loxp cassette followed by the ASM encoding cDNA (Smpd1) (25). Breeding of these mice with CD4cre mice results in T cell-specific excision of the STOP cassette and enforced ASM expression. To confirm elevated ASM expression in T cells from t-ASM/CD4cre double-transgenic mice, we analyzed the ASM activity of unstimulated and anti-CD3/anti-CD28 stimulated T cells isolated from spleen of t-ASM/CD4cre (TG) and t-ASM controls (WT). CD4^+^ and CD8^+^ naïve T cells from t-ASM/CD4cre mice exhibited elevated ASM activity compared to T cells isolated from WT littermates. Stimulation of T cells with anti-CD3/anti-CD28 resulted in an increase of ASM activity in CD4^+^ and CD8^+^ T cells from WT controls. This induction in ASM activity upon stimulation was strongly enhanced in T cells isolated from t-ASM/CD4cre mice, which showed approximately 10 fold higher ASM activity than stimulated T cells from littermate controls (Figure 1A). Stimulation of CD19^+^ B cells with LPS also resulted in elevated ASM activity. However, we did not observe differences in ASM activity between unstimulated or LPS-stimulated CD19^+^ B cells isolated from t-ASM/CD4cre mice compared to control mice (Figure 1A). Hence, t-ASM/CD4cre mice are a suitable model to analyze the impact of ASM activity on T cell phenotype and function.

First, we asked whether enhanced T cell-specific ASM activity has an impact on the development of T cells. Therefore, we determined the frequencies of CD4^+^ T cells, CD8^+^ T cells, and Foxp3^+^ Tregs of CD4^+^ T cells in thymus and spleen isolated from t-ASM/CD4cre mice and WT controls. Whereas, we did not observe any differences in the percentage of CD4^+^ T cells and CD8^+^ T cells (Figure 1B), T cell-specific overexpression of ASM resulted in a significant reduction of Foxp3-expressing Tregs in the spleen of t-ASM/CD4cre mice compared to controls (Figure 1C).

### ASM Overexpression Impairs Treg Differentiation and Improves Th1 Differentiation *in vitro*

**Figure 2:**
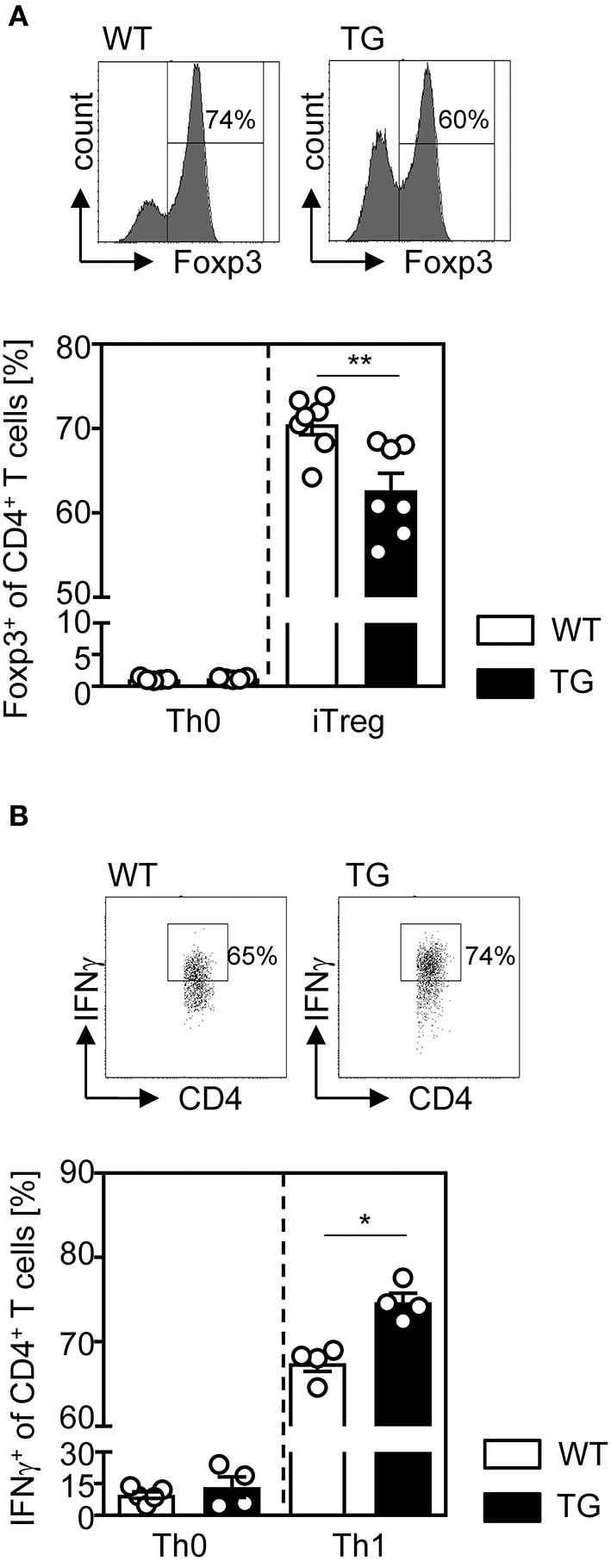
Impaired regulatory T cell differentiation and elevated induction of Th1 cells from ASM-overexpressing CD4^+^ T cells under respective polarizing conditions *in vitro*. **(A)** Sorted CD4^+^CD25^−^ T cells from t-ASM/CD4cre mice (TG) and t-ASM littermates (WT) were stimulated with anti-CD3 and anti-CD28 and differentiated into Foxp3^+^ Tregs in the presence of TGF-β and IL-2 for 3 days or **(B)** into IFN-γ producing Th1 cells, by adding IL-12, and anti-IL-4 to the cells for 6 days. Respective controls (Th0) were only stimulated with anti-CD3 and anti-CD28. Data from two to three independent experiments with *n* = 6–7 mice in total are shown as mean ± SEM. *p < 0.05, **p < 0.01.

Since we observed decreased frequencies of Tregs in the spleen, but not in the thymus of t-ASM/CD4cre mice (Figure 1C), we next analyzed the efficacy of ASM-overexpressing T cells to differentiate into induced Tregs (iTregs) *in vitro*. For this purpose, we isolated CD4^+^CD25^−^ T cells from t-ASM/CD4cre mice as well as WT littermates and stimulated them with anti-CD3/anti-CD28 in the presence (iTreg) or absence (Th0) of IL-2 and TGF-β. As depicted in Figure 2A, T cell-specific overexpression of ASM resulted in significantly reduced induction of Foxp3^+^ iTregs compared to WT controls (Figure 2A). In addition, we investigated the capacity of CD4^+^CD25^−^ T cells to differentiate into IFN-γ producing Th1 cells *in vitro*. CD4^+^CD25^−^ T cells were isolated from T cell-specific ASM-overexpressing mice and WT littermates, stimulated with anti-CD3/anti-CD28 and treated with IL-12 and anti-IL-4 (Th1). As control, T cells were stimulated without adding IL-12 and anti-IL-4 (Th0). Enforced ASM expression resulted in a significantly increased induction of IFN-γ producing T cells upon stimulation under Th1 polarizing conditions (Figure 2B).

### ASM Overexpressing T Cells Exhibit Increased Proliferative Activity and T Cell Receptor Signaling *in vitro*

**Figure 3:**
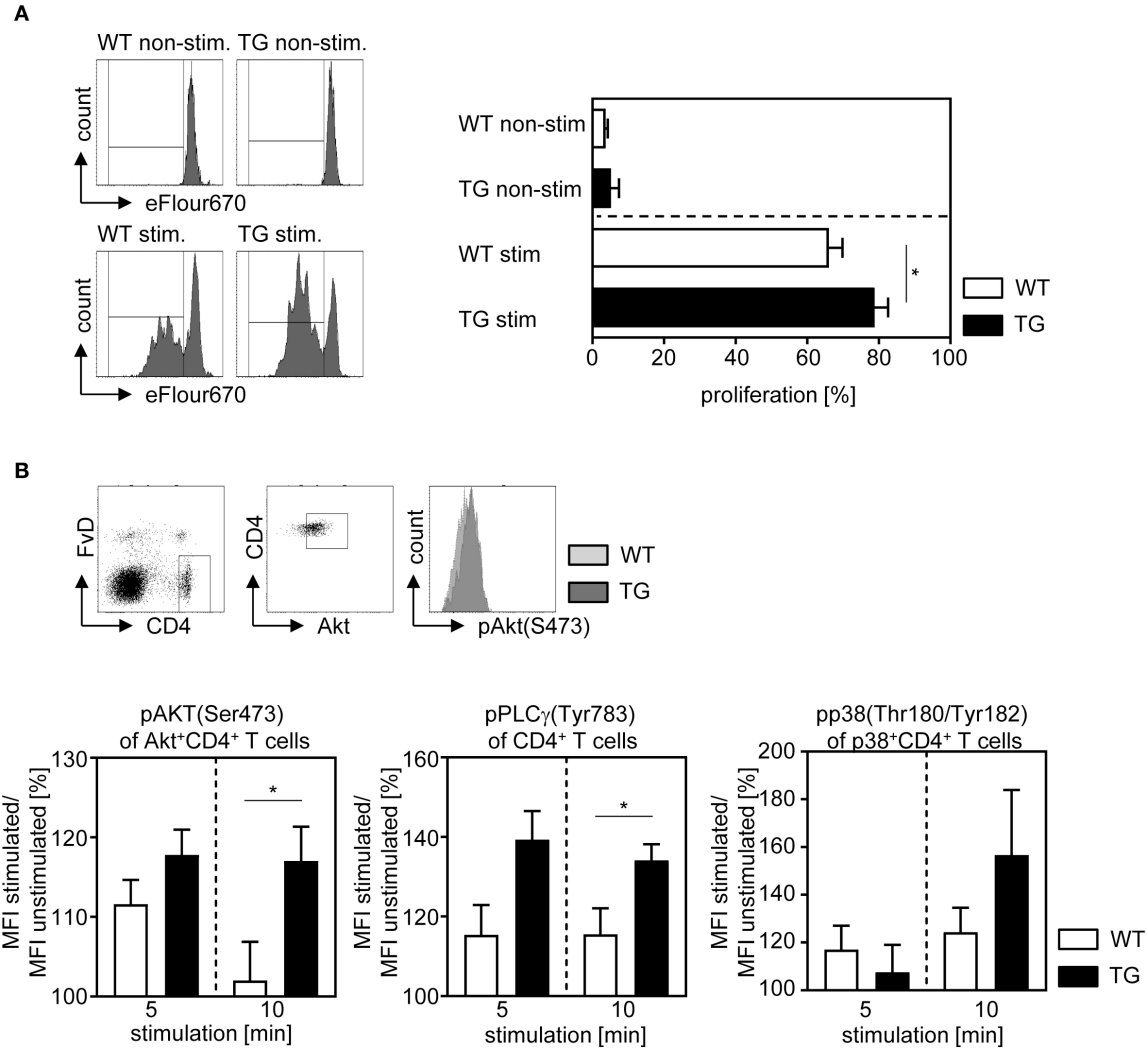
Overexpression of ASM in T cells enhances the proliferative activity and T cell receptor signaling. **(A)** CD4^+^CD25^−^ T cells were isolated from t-ASM/CD4cre mice (TG) or t-ASM controls (WT), labeled with cell proliferation dye eFluor 670 and stimulated for 72 h with anti-CD3 and anti-CD28. Proliferation was assessed as loss of cell proliferation dye eFluor 670 by flow cytometry. **(B)** Splenocytes were isolated from t-ASM/CD4cre mice (TG) or t-ASM controls (WT), left unstimulated or stimulated with anti-CD3 and anti-CD28 for 5 or 10 min and analyzed for phospho-Akt of gated Akt^+^CD4^+^ T cells, phospho-PLCγ1 of gated CD4^+^ T cells and phospho-p38 of gated p38^+^CD4^+^ T cells by flow cytometry. The gating strategy and a representative histogram-overlay of Akt phosphorylation of stimulated CD4^+^ T cells from t-ASM/CD4cre mice (TG) and t-ASM controls (WT) are shown in the upper panel. The increase in phosphorylation was calculated as percentage MFI stimulated/ MFI unstimulated. Results from two to three independent experiments with *n* = 5–9 mice in total are summarized as mean ± SEM. *p < 0.05.

For analyzing whether ASM expression has also an impact on the proliferative response of T cells, we isolated CD4^+^CD25^−^ T cells from naïve t-ASM/CD4cre mice and control littermates, labeled them with the cell proliferation dye eFluor 670 and left them untreated or stimulated them with anti-CD3 and anti-CD28 for 3 days *in vitro*. As expected, unstimulated T cells showed only very low proliferative activity with no differences between ASM-overexpressing cells and controls. However, CD4^+^CD25^−^ T cells from t-ASM/CD4cre mice exhibited significant elevated proliferation compared to T cells from WT littermates upon stimulation *in vitro* (Figure 3A). To gain further insights into the underlying processes, we investigated the activation of the TCR signaling pathway in more detail. Upon TCR activation a cascade of several signaling molecules, including PLCγ, Akt and p38 is phosphorylated (27). To analyze the impact of T cell-intrinsic ASM expression on TCR signaling, we isolated splenocytes from t-ASM/CD4cre mice and respective littermates, stimulated them with anti-CD3/anti-CD28 and analyzed the phosphorylation of Akt, PLCγ, and p38 MAPK on gated CD4^+^Akt^+^, CD4^+^, or CD4^+^p38^+^ T cells, respectively at different time points by flow cytometry. As expected, we detected an increased phosphorylation of all three molecules in WT and ASM-overexpressing T cells upon activation. Strikingly, enforced expression of ASM in T cells resulted in elevated phosphorylation of Akt, PLCγ, and p38 upon TCR stimulation *in vitro* (Figure 3B). These results indicate that anti-CD3/anti-CD28 stimulation induces ASM activity which in turn influences the TCR signaling pathway and thereby the proliferation and differentiation of CD4^+^ T cells *in vitro*.

### T-ASM/CD4cre Mice Show Enhanced T Cell Proliferation and IFN-γ Production in Response to *P. yoelii* Infection

**Figure 4:**
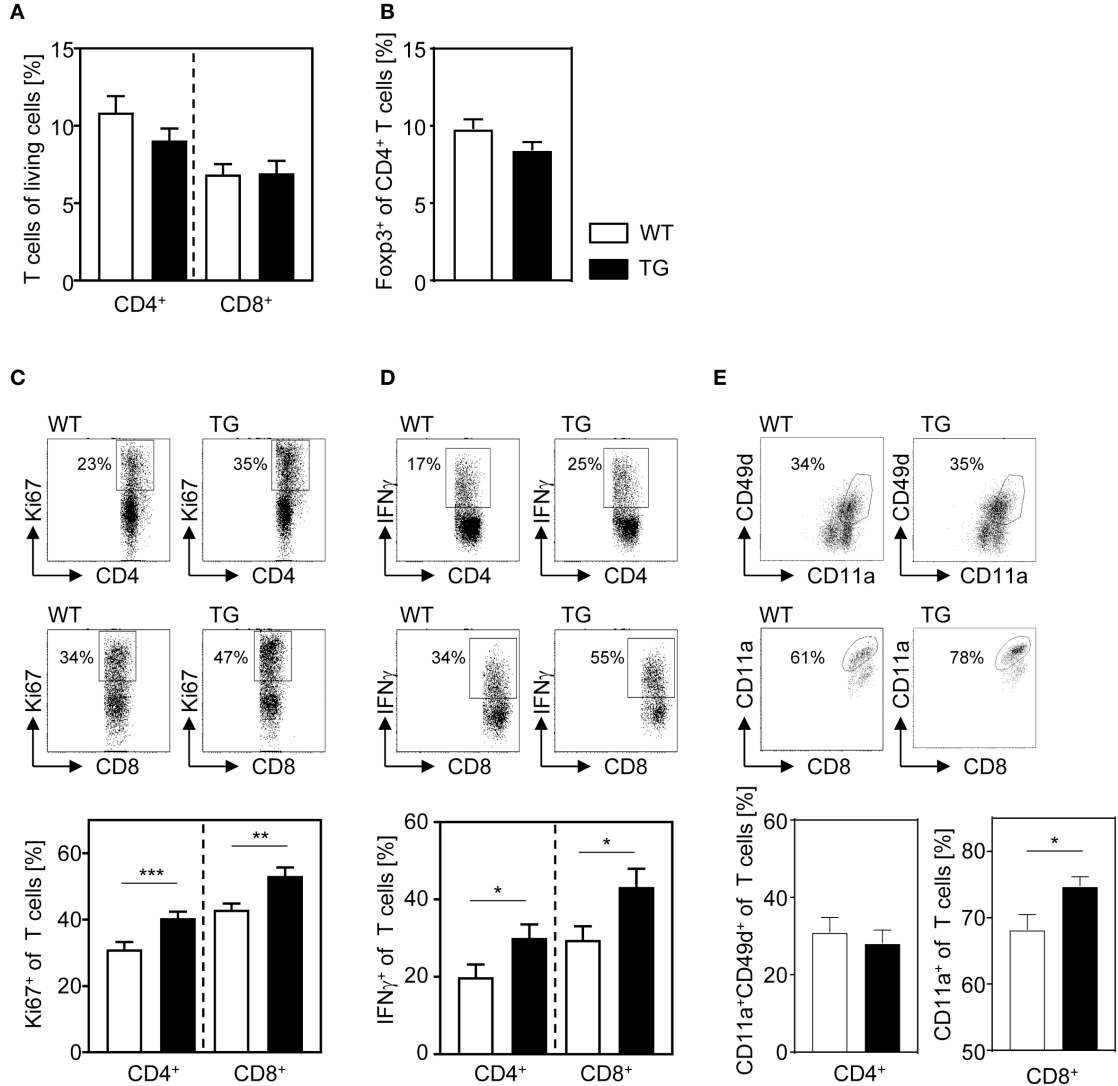
T cell-specific overexpression of ASM results in enhanced T cell activation after *P. yoelii* infection. T-ASM/CD4cre mice (TG) and t-ASM littermates (WT) were infected with *P. yoelii* and analyzed at day 14 post infection. **(A)** The frequency of CD4^+^ T cells and CD8^+^ T cells, **(B)** Foxp3^+^ regulatory T cells of CD4^+^ T cells, **(C)** Ki67- expressing, and **(D)** IFN-γ producing CD4^+^ T cells and CD8^+^ T cells as well as the percentage of **(E)** CD11a^+^CD49d^+^ CD4^+^ T cells and CD11a-expressing CD8^+^ T cells from spleen of *P. yoelii*-infected t-ASM/CD4cre mice (TG) and t-ASM littermates (WT) were determined by flow cytometry. Representative dot blots are shown in the upper panel of **(C–E)**. Results from at least three independent experiments with *n* = 14–19 mice in total are summarized as mean ± SEM. *p < 0.05, **p < 0.01, ***p < 0.001.

Our data show that T cell-specific overexpression of ASM enhances T cell activation upon stimulation *in vitro*. Therefore, we next asked whether enforced ASM activity in T cells also results in elevated immunity in the more complex *in vivo* situation. For this purpose, we infected t-ASM/CD4cre mice and WT control littermates with *P. yoelii*. At day 14 post infection (p.i.), we analyzed the phenotype of T cells from spleen of infected mice by flow cytometry. As depicted in Figures 4A,B, the frequencies of CD4^+^ T cells, CD8^+^ T cells, as well as Foxp3^+^ Tregs of CD4^+^ T cells did not differ between *P. yoelii*-infected T cell-specific ASM-overexpressing mice and control littermates (Figures 4A,B). Well in line with our *in vitro* data, we detected significantly elevated frequencies of proliferated CD4^+^ T cells as well as CD8^+^ T cells in t-ASM/CD4cre mice compared to WT controls as measured by Ki67 expression (Figure 4C). Moreover, the percentage of IFN-γ expressing CD4^+^ T cells and CD8^+^ T cells was significantly increased in T cell-specific ASM overexpressing mice upon *P. yoelii* infection in comparison to WT littermates (Figure 4D). To gain further insights into the impact of ASM expression on *Plasmodium*-specific T cell responses, we analyzed the frequencies of CD11a^+^CD49d^+^ CD4^+^ T cells as well as the percentage of CD11a-expressing CD8^+^ T cells. This approach was already described for the detection of antigen-specific T cells during *Plasmodium* infection (28). We did not observe differences in the percentages of CD11a^+^CD49d^+^ CD4^+^ T cells between T cell-specific ASM overexpressing mice and control littermates at least at day 14 p.i. (Figure 4E). However, *P. yoelii* infection of t-ASM/CD4cre mice resulted in significantly increased frequencies of antigen-experienced CD8^+^ T cells in comparison to WT littermates. Together, these data suggest that T cell-intrinsic ASM activity modulates T cell activation during an ongoing immune response *in vivo*.

### Enhanced Systemic Pro-Inflammatory Cytokine Production and Reduced Parasitemia in *P. yoelii*-Infected t-ASM/CD4cre Mice

**Figure 5:**
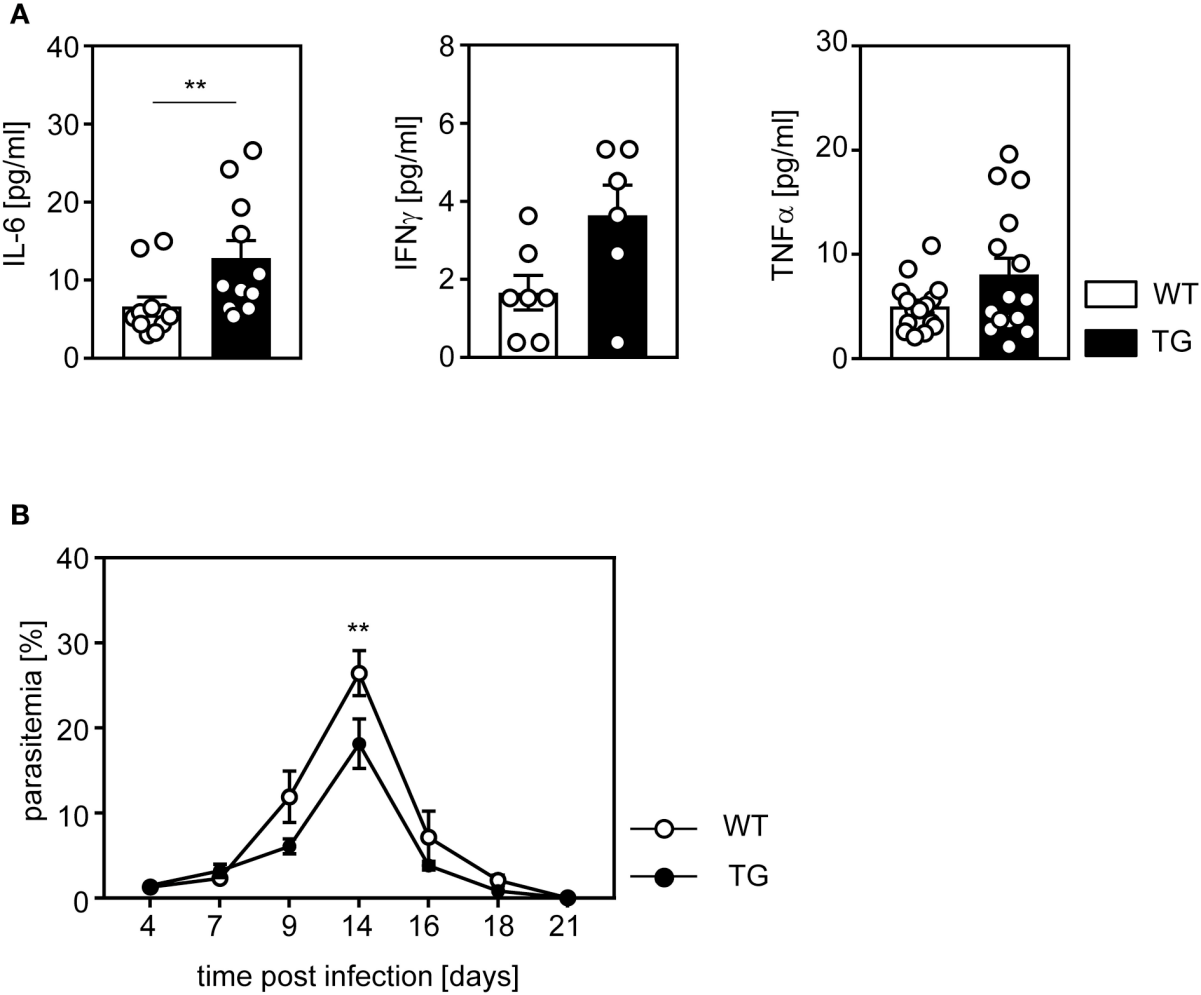
Elevated IL-6 serum levels and improved parasite clearance in *P. yoelii*-infected t-ASM/CD4cre mice. T-ASM/CD4cre mice (TG) and t-ASM littermates (WT) were infected with *P. yoelii*. **(A)** IL-6, IFN-γ, and TNF-α serum levels were determined by Luminex technology at day 14 p.i. **(B)** Parasitemia was determined at indicated time points post infection by Giemsa staining. Results from two to four independent experiments with *n* = 6–15 mice in total are summarized as mean ± SEM. **p < 0.01.

To further investigate the impact of ASM-dependent increased T cell activation on the course of *P. yoelii* infection, we determined the amount of pro-inflammatory cytokines in serum of t-ASM/CD4cre mice and WT littermates at day 14 post infection (p.i.). *P. yoelii*-infected T cell-specific ASM-overexpressing mice exhibited a significant increase in systemic production of IL-6, as well as IFN-γ and TNF-α, although statistically not significant, in comparison to control mice (Figure 5A). Finally, we asked whether the elevated T cell response in t-ASM/CD4cre mice influences the parasitemia upon *P. yoelii* infection. For this purpose we determined the percentage of infected RBCs at day 4, 7, 9, 14, 16, 18, and 21 p.i. by Giemsa-stained blood smears and detected significantly less parasitized RBCs in t-ASM/CD4cre mice than in WT littermates at day 14 p.i. (Figure 5B). In summary, these results indicate that enforced ASM expression in T cells results in elevated T cell activation in *P. yoelii*-infected mice accompanied by improved parasite clearance.

## Discussion

The enzyme ASM hydrolyzes sphingomyelin to ceramide upon activation by several cellular stress signals (29). This results in the formation of ceramide-rich platforms within the cell membrane which have an impact on different cellular processes. It was already proposed that ASM also influences activation, differentiation, and stability of CD4^+^ T cell subsets. However, these studies either addressed the impact of ASM on whole CD4^+^ T cells (9, 10, 14) or focused on Tregs (12, 13) isolated from ubiquitous ASM-deficient mice or upon treatment with pharmacological inhibitors of ASM. Here, we made use of T cell-specific ASM overexpressing mice to clarify the T cell-intrinsic effect of ASM activity on T cells in more detail. By this approach we can exclude an indirect effect of ASM expressed by other cells or side effects of pharmacological ASM inhibitor treatment such as inhibition of other enzymes involved in the sphingolipid metabolism.

Our data indicate that T cell-specific overexpression of ASM results in significantly reduced relative number of Foxp3^+^ Tregs within the spleen (Figure 1C). Well in line, ASM-deficient mice showed an increase in Treg numbers (12, 13). Interestingly, we detected no differences in the percentage of Tregs within the thymus, which suggests that T cell-intrinsic ASM activity does not influence the development of thymus-derived Tregs *per se*, but rather their induction and/ or stability in the periphery. Hollmann and colleagues claimed that Tregs are more resistant to ASM-deficiency than CD4^+^Foxp3^−^ T cells, since they observed a reduction in absolute cell numbers of CD4^+^Foxp3^−^ non-Tregs, but no differences in CD4^+^CD25^+^Foxp3^+^ Treg numbers in Asm-deficient mice and after treatment of mice with amitriptyline (13). In contrast, Zhou et al., detected increased relative as well as absolute numbers of CD4^+^CD25^+^Foxp3^+^ Tregs in spleen of ASM-deficient mice (12). We demonstrate that sorted ASM-overexpressing CD4^+^CD25^−^ T cells differentiate less efficiently into Foxp3^+^ Tregs upon stimulation in the presence of IL-2 and TGF-β *in vitro* than WT controls (Figure 2A). Similar results were also obtained from ASM-deficient T cells, which expressed more Foxp3 than WT cells under Treg polarizing conditions *in vitro* (12). Together these results provide evidence that T cell-intrinsic ASM activity is involved in the *de novo* induction of iTregs rather than in the intrathymic development.

To gain further insights into this process, we analyzed the phosphorylation of different TCR signaling molecules. As expected, we observed an increase in p-Akt(Ser473), p-p38 and pPLCγ-1 upon anti-CD3/anti-CD28 stimulation of WT and ASM-overexpressing cells (Figure 3B). However, stimulated CD4^+^ T cells from t-ASM/CD4cre mice exhibited elevated phosphorylation of these molecules in comparison to stimulated WT cells. Similar results were also described for human CD4^+^ T cells. Pharmacological inhibition of ASM by imipramine resulted in decreased phosphorylation of PLCγ and Akt upon anti-CD3/anti-CD28 stimulation (14), linking ASM activity to mediators of CD4^+^ T cell signals and activation. Ser473-phosphorylated Akt has been described to preferentially phosphorylate Foxo1 and Foxo3 (30), which results in their retention within the cytoplasm (31). Nuclear exclusion of Foxo proteins prevents direct binding to the Foxp3 promotor (32, 33) and thereby interferes with Foxp3 expression. Along with elevated Akt activation, we also detected increased phosphorylation of p38. This signaling pathway also seems to negative regulate the induction of iTregs. P38-deficient T cells showed attenuated MAPK-activated proteinkinase-dependent mTOR signaling after TCR stimulation accompanied by enhanced differentiation into iTregs under appropriate polarizing conditions (34). On the other hand, p38 is also involved in the regulation of IFN-γ expression. By using both, pharmacological inhibitors and genetically modified mice, it has been shown that the p38 MAPK pathway is required for the production of IFN-γ and Th1 differentiation (35). Well in line, we demonstrated that CD4^+^CD25^−^ T cells from t-ASM/CD4cre mice differentiate more efficiently into IFN-γ producing Th1 cells than WT cells upon TCR engagement in the presence of IL-12 and anti-IL-4 (Figure 2B). These results correlate with decreased Th1 differentiation of human CD4^+^ T cells upon treatment with the ASM-inhibitor imipramine (14). Hence, the ASM-dependent increase in Akt and p38 phosphorylation upon TCR engagement might be responsible for the observed less effective induction of Foxp3^+^ iTregs and elevated differentiation of stimulated CD4^+^ T cells into Th1 cells under respective polarizing conditions. Activation of TCR signaling pathways upon anti-CD3/anti-CD28 stimulation have also an impact on T cell proliferation (36). Splenocytes from ASM-deficient mice and human CD4^+^ T cells treated with pharmacological ASM-inhibitors showed reduced proliferation upon activation (10, 14). Well in line, we detected significant elevated proliferation of stimulated ASM-overexpressing CD4^+^CD25^−^ T cells compared to WT controls (Figure 3A), providing evidence that T cell-intrinsic ASM activity directly influences the proliferative activity of T cells.

To clarify whether enforced T cell-intrinsic ASM activity has also an impact on T cell function during an ongoing immune response *in vivo*, we infected t-ASM/CD4cre mice and control littermates with *P. yoelii*. It is well established that in addition to B cells, CD4^+^ and CD8^+^ T cells as well as IFN-γ play an important role in this mouse model for blood stage malaria (17). According to our *in vitro* analysis, we detected elevated relative numbers of proliferating and IFN-γ producing T cells (Figure 4). Strikingly, T cell-specific ASM overexpressing mice exhibited significantly less parasitemia than WT mice (Figure 5B), indicating that enforced ASM activity in T cells contributes to elevated T cell activation *in vivo*. Interestingly, infection of ASM-deficient or amitriptyline-treated mice with *P. berghei*, which causes experimental cerebral malaria, also resulted in decreased parasitemia (37). In that study the authors did not analyze the phenotype of T cells, but speculated about an impact of ASM on release or invasion of parasites from or into erythrocytes, respectively (37), which are not affected in T cell-specific ASM-overexpressing mice used in this study. We have shown that Foxp3^+^ Tregs dampen an effective immune response resulting in impaired pathogen clearance in *P. yoelii*-infected mice (20). Interestingly, we did not detect significant differences in the relative number of Foxp3^+^ Tregs in *P. yoelii*-infected T cell-specific ASM-overexpressing mice compared to WT controls. We could not exclude that t-ASM/CD4cre mice have decreased Treg numbers at early time points of infection, which might impact the course of infection. However, our *in vitro* analysis indicate that ASM-overexpressing CD4^+^ T cells exhibit enhanced proliferative activity and differentiate more effectively into IFN-γ producing Th1 cells than WT cells, even in the absence of Tregs.

Overall, our results indicate that T cell-intrinsic ASM activity plays an important role during T cell proliferation and differentiation into iTreg and Th1 CD4^+^ T cell subsets. Hence, the ASM/sphingolipid pathway might be a novel target for the therapy of T cell-dependent diseases.

## Data Availability

The raw data supporting the conclusions of this manuscript will be made available by the authors, without undue reservation, to any qualified researcher.

## Ethics Statement

This study was carried out in accordance with the recommendations of the Society for Laboratory Animal Science (GV-SOLAS) and the European Health Law of the Federation of Laboratory Animal Science Associations (FELASA). The protocol was approved by the state authority for nature, environment and customer protection (LANUV), North Rhine-Westphalia, Germany.

## Author Contributions

MH, AG, HA, SB, MK, and KB designed and performed the experiments and analyzed data. JB and AW were involved in data discussion and in drafting the manuscript. WH initiated, organized and designed the study. MH and WH wrote the manuscript.

### Conflict of Interest Statement

The authors declare that the research was conducted in the absence of any commercial or financial relationships that could be construed as a potential conflict of interest.
